# Overexpression of the elongation factor *MtEF1A1* promotes salt stress tolerance in *Arabidopsis thaliana* and *Medicago truncatula*

**DOI:** 10.1186/s12870-023-04139-5

**Published:** 2023-03-13

**Authors:** Lei Xu, Lixia Zhang, Yajiao Liu, Bilig Sod, Mingna Li, Tianhui Yang, Ting Gao, Qingchuan Yang, Ruicai Long

**Affiliations:** 1 State Key Laboratory of Grassland Agro-ecosystems, Key Laboratory of Grassland Livestock Industry Innovation, Ministry of Agriculture and Rural Affairs, Engineering Research Center of Grassland Industry, Ministry of Education, College of Pastoral Agriculture Science and Technology, Lanzhou University, Lanzhou, 730000 China; 2 Institute of Animal Sciences, Chinese Academy of Agricultural Sciences, Beijing, 100000 China; 3 Institute of Animal Sciences, Ningxia Academy of Agriculture and Forestry Sciences, Yinchuan, 750000 China

**Keywords:** *Medicago truncatula*, Elongation factor, Transgenic, Salt stress, Calmodulin

## Abstract

**Background:**

Elongation factor 1 A (*EF1A*), an essential regulator for protein synthesis, has been reported to participate in abiotic stress responses and environmental adaption in plants. However, the role of *EF1A* in abiotic stress response was barely studied in *Medicago truncatula*. Here, we identified elongation factor (*EF*) genes of *M. truncatula* and studied the salt stress response function of *MtEF1A1* (MTR_6g021805).

**Results:**

A total of 34 *EF* genes were identified in the *M. truncatula* genome. Protein domains and motifs of *EFs* were highly conserved in plants. *MtEF1A1* has the highest expression levels in root nodules and roots, followed by the leaves and stems. Transgenic *Arabidopsis thaliana* overexpressing *MtEF1A1* was more resistant to salt stress treatment, with higher germination rate, longer roots, and more lateral roots than wild type plant. In addition, lower levels of H_2_O_2_ and malondialdehyde (MDA) were also detected in transgenic *Arabidopsis*. Similarly, *MtEF1A1* overexpressing *M. truncatula* was more resistant to salt stress and had lower levels of reactive oxygen species (ROS) in leaves. Furthermore, the expression levels of abiotic stress-responsive genes (*MtRD22A* and *MtCOR15A*) and calcium-binding genes (*MtCaM* and *MtCBL4*) were upregulated in *MtEF1A1* overexpressing lines of *M. truncatula*.

**Conclusion:**

These results suggested that *MtEF1A1* play a positive role in salt stress regulation. *MtEF1A1* may realize its function by binding to calmodulin (CaM) or by participating in Ca^2+^-dependent signaling pathway. This study revealed that *MtEF1A1* is an important regulator for salt stress response in *M. truncatula*, and provided potential strategy for salt-tolerant plant breeding.

**Supplementary Information:**

The online version contains supplementary material available at 10.1186/s12870-023-04139-5.

## Background

Elongation factors (EFs) are common cellular proteins that are responsible for the extension of peptide chains during protein synthesis. There are three prokaryotic extension factors: EF thermo unstable (EF-Tu), EF thermo stable (EF-Ts), and elongation factor G (EF-G). Eukaryotic EFs can be divided into the eEF1, eEF2, and eEF3 families. The eEF1 complex causes conformational changes in ribosomes and catalyzed aa-tRNA, resulting in the hydrolysis of GTP and the release of eEF1A·GDP. eEF1 contains four subunits: eEF1A, eEF1Bα, eEF1Bβ, and eEF1Bγ [1]. eEF1A is equivalent to prokaryotic EF-Tu, accounting for 1–2% of total cellular protein content [2]. The guanine nucleotide exchange factor (GEF) eEF1B promotes the exchange of GDP and GTP, allowing eEF1A to participate in another round of peptide chain extension [3]. eEF1Bα is also involved in plant phosphorylation and is related to serine [4]. In *Arabidopsi*s, the silencing of eEF1Bβ leads plant dwarfing and reduced total lignin and crystalline cellulose, respectively [5]. eEF1Bγ has been demonstrated to participate in oxidative stress response and may be related to its glutathione transferase (GST) properties [6]. eEF2 is a monomer that catalyzes the movement of ribosomes bound to mRNA. Through the phosphorylation and inactivation of eukaryotic extension factor-2 kinase (eEF2K), extension of peptide chains is hindered and protein synthesis was inhibited [7]. In eukaryotes, eEF3 can assist eEF1A delivering aa-tRNA and removing deacyl-tRNA from the E-site, ensuring peptide chain extension [8]. Together with two paralogous genes, YEF3 (YLR249W) and HEF3 (YNL014W), eEF3 also plays an important role in detoxification of reactive oxygen species (ROS) [8].

*Arabidopsis thaliana* contains a family of four *EF1A* genes (*AtEF1A1*–*4*) that are divided into two subfamilies based on sequence similarity and physical location within the genome [9]. The four *EF1A* genes in soybean (*Glycine max*) can also be divided into two subfamilies, type A and type B; Al and A2 are highly expressed in green organs (leaves and stems), whereas there is little difference in expression levels of the four genes in the roots [10]. Eukaryotic cells contain two isoforms of *EF1A*, *EF1A1* and *EF1A2*, that have structural differences [11]. Some studies have shown that the structure and sequences of plant *EF1A* genes are relatively conserved across species. It has been previously reported that *AtEF1A2* (AT1G07930) and *AtEF1A4* (AT5G60390) have stable structures and high expression levels in *Arabidopsis* [12, 13]. Analysis with NormFinder, which compares potential internal reference genes, showed that *EF1A* was expressed stably under pyrene and heavy metal treatments in mangrove (*Aegiceras corniculatum*) [14]. Furthermore, *EF1A* was stably expressed under salt, osmotic, and metal stress conditions in cucumber (*Cucumis sativus*) [15] and under drought stress in Chinese cabbage (*Brassica rapa*) [15, 16].

*EF1A* genes are induced by environmental stress in many plants. *EF1A* expression was found to be induced by heat and cold stress in barley (*Hordeum vulgare*) and maize (*Zea mays*) [17–19]. In wheat (*Triticum aestivum*), *EF1A* expression increases in response to heat; this may be due to the involvement of *EF1A* in protein folding as a molecular chaperone, protecting heat-resistant proteins from heat damage or enhancing heat resistance by promoting protein synthesis or transcriptional activation [20, 21]. Subsequent studies have found that two or three basic peptides or subtypes of *EF1A* can increase *EF1A* abundance under heat stress [22]. Yeast hybrid and northern blot assays showed that *AcEF1A* of *Bruguiera sexangula* enhances tolerance to salt and osmotic stress in *Escherichia coli* [23]. A cDNA clone of *AtEF1A2* was isolated from a salt-sensitive calcineurin (CaN) yeast deletion mutant library. This gene has companion protein activity promoting refolding of unfolded proteins in cell or in vitro which enhances salt tolerance in yeast, and overexpression in plants confers salt tolerance [24]. In recent years, researchers have studied the regulatory network of miRNAs responsive to salt stress in barley; they found that *EF1A* plays a role in salt tolerance as a molecular companion and is the target gene of miR169i and the novel miRNA PC-mir124 [25].

Plants respond and adapt to salt stresses by osmotic stress and ion stress, which can reduce the cell volume and the cell division rate, slow the growth of leaves and roots at different stages, and cause inhibition of plant photosynthesis [26, 27]. Salt stress accumulation can also promote the accumulation of reactive oxygen species (ROS) and damage the cell membrane structure [28]. Compared with the control group, the overexpression of soybean *GmEF4* significantly delayed leaf wilting, prolonged root system, increased biomass and decreased ROS accumulation under drought and salt treatment [29]. Meanwhile, *EF1A* has rarely been reported to be involved in *Medicago truncatula* salt stress signaling. In our previous study, we conducted an iTRAQ proteomic analysis in *M. truncatula* ecotype R108 and *M. sativa* cv. Zhongmu3. From those data, an elongation factor protein (MTR_6g021805) was found to be induced by salt stress (1.51-fold and 1.20-fold change in *M. truncatula* and *M. sativa*, respectively) [30, 31]. Here, we cloned *MtEF1A1* from *M. truncatula* and explored the molecular mechanisms through which it participates in salt stress responses. We found that *MtEF1A1* overexpression enhanced salt tolerance in *Arabidopsis* and *M. truncatula*, and a model of *MtEF1A1* regulation under salt stress was proposed, likely through calcium signaling pathways. These results position *MtEF1A1* as a promising candidate gene for breeding salt tolerance in forage plants.

## Results

### Structural analysis of ***MtEF1A1***

**Fig. 1:**
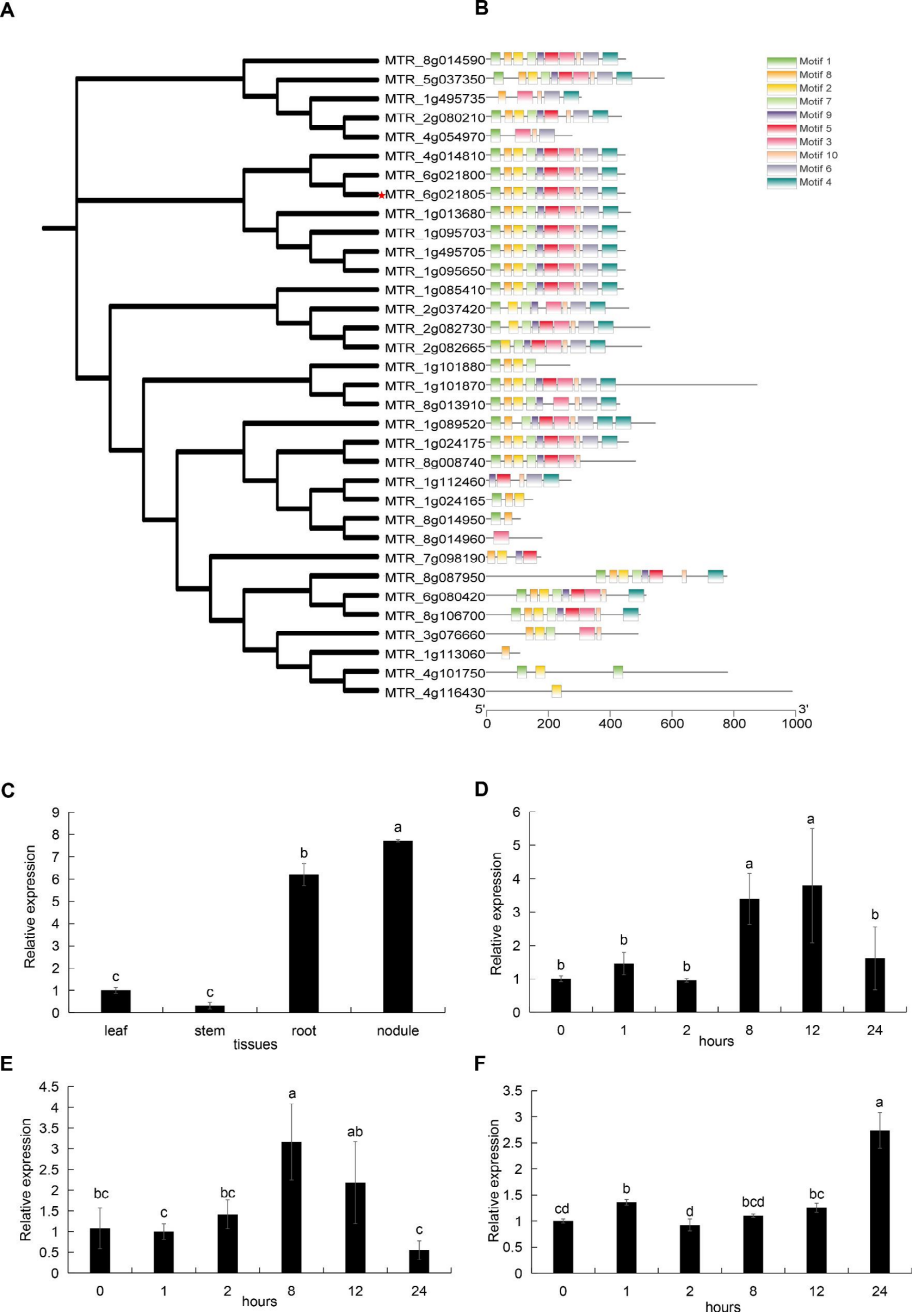
Bioinformatic analyses of *EF* genes in *M. truncatula* and expression pattern of *MtEF1A1.* (**A**) Phylogenetic tree shows the relationships between the 34 *EF* gene family members in *M. truncatula*. *MtEF1A* (MTR_6g021805) is marked by a red pentagram. (**B**) Analysis of conserved motifs in *EF* family proteins. The conserved motifs 1–10 were indicated by different colors. (**C**) Expression levels of *MtEF1A1* of different tissues in wild-type *M. truncatula* (R108) and expression levels were normalized to leaf. Different lowercase letters indicate significant differences (*p* < 0.05). Differential expression of *MtEF1A1* in response to 200 mM NaCl for 0, 1, 2, 8, 12, 24 h in *M. truncatula* R108 of leaf (**D**), stem (**E**) and root (**F**). Each tissues expression level in salt-treated was normalized to that of plants at point 0 h. Values are means ± SD of three biological replicates. Different lowercase letters indicate significant differences (*p* < 0.05)

*MtEF1A1* has a 447-bp coding sequence (CDS), which encodes a 49.24-kDa protein with a predicted isoelectric point (*pI*) of 8.02. EF1A has three conserved domains: GTP_EFTU (Pfam: PF00009), GTP_EFTU_D2 (Pfam: PF03144), and GTP_EFTU_D3 (Pfam: PF03143). There are 34 members of the *MtEF* gene family (Fig. 1A). Protein structure analysis showed that 14 of the 34 EF family genes contained 10 motifs, and the remaining 20 proteins had 1–9 motifs (Fig. 1B). Expression Logo of motifs in *EF* genes in *M. truncatula* were shown in Fig. S2. The amino acid sequence of *MtEF1A1* was aligned with EF1A proteins from *Arabidopsis*, soybean, and rice (*Oryza sativa*). There was > 94% sequence similarity between *MtEF1A1, GmEF1A* (GLYMA_17G186600), *OsEF1A* (Os03g0177400), and *AtEF1A1*–*4* (AT1G07920, AT1G07930, AT1G07940, and AT5G60390), indicating that *EF1A* was highly evolutionarily conserved. *MtEF1A1* had four conserved regions (G-1 to G-4) predicted to be involved in GDP/GTP exchange and GTP hydrolysis based on sequence comparison to proteins in other species (Additional file 2: Fig. S1A). The average hydrophilic coefficient was − 0.645. *MtEF1A1* was found to be a hydrophilic protein with no transmembrane structure or signal peptide (Additional file 2: Fig. S1B), and was predicted to be localized to the cytoplasm and nucleus. Tertiary protein structure of MtEF1A1 was predicted (Additional file 2: Fig. S1C). Random coils (37.14%) and alpha helixes (29.53%) were the dominant predicted secondary structures (Additional file 2: Fig. S1D).

### ***MtEF1A1*** was salt stress-inducible

Quantitative real-time RT-PCR was used to detect detailed changes in *MtEF1A1* expression in different tissues or response to salt treatment. By analyzing the expression levels in different tissues, the results showed that *MtEF1A1* was expressed at higher levels in the root nodules than in the roots of *M. truncatula* (Fig. 1C). The results in response to salt treatment showed that *MtEF1A1* was induced by salt stress in multiple tissues, and the expression level increased significantly after 8 h (Fig. 1D-F). In untreated plants, *MtEF1A1* expression was highest in the roots, followed by the leaves and stems (Fig. 1C).

### ***MtEF1A1*** overexpression improved salt tolerance in transgenic ***Arabidopsis***

**Fig. 2:**
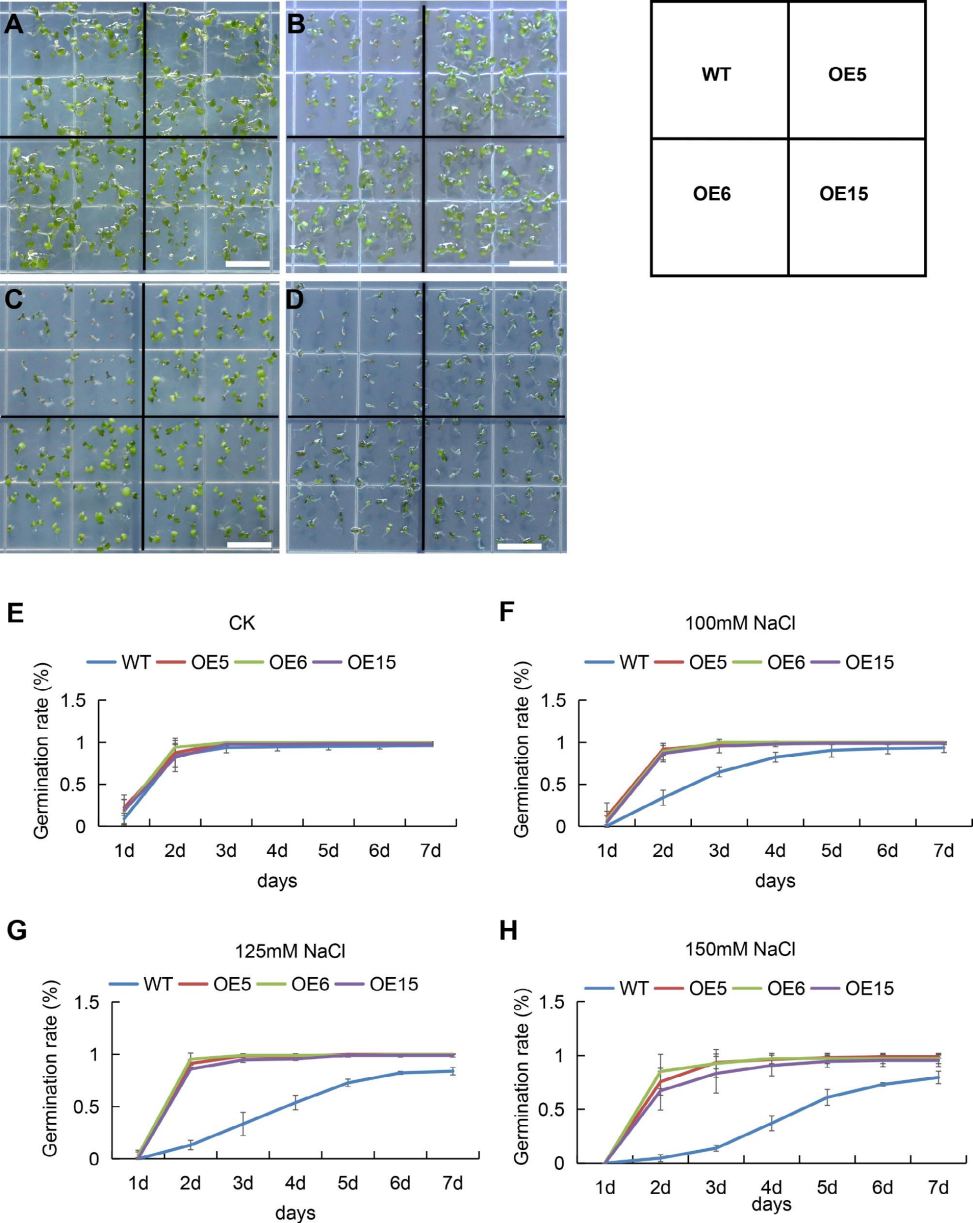
Germination of wild-type (WT) *Arabidopsis* plants and those overexpressing *MtEF1A1*. (**A-D**) Germination and growth of WT and *MtEF1A1*-overexpression (OE) *Arabidopsis* seedlings on medium containing NaCl at 0 mM (**A**), 100 mM (**B**), 125 mM (**C**), and 150 mM for 7 days (**D**). Scale bar = 1 cm. CK, control (0 mM NaCl). (**E-H**) Germination rates of WT and *MtEF1A1*-OE seeds sown on medium containing NaCl at 0 mM (**E**), 100 mM (**F**), 125 mM (**G**), and 150 mM (**H**). Values are means ± SD of three biological replicates

**Fig. 3:**
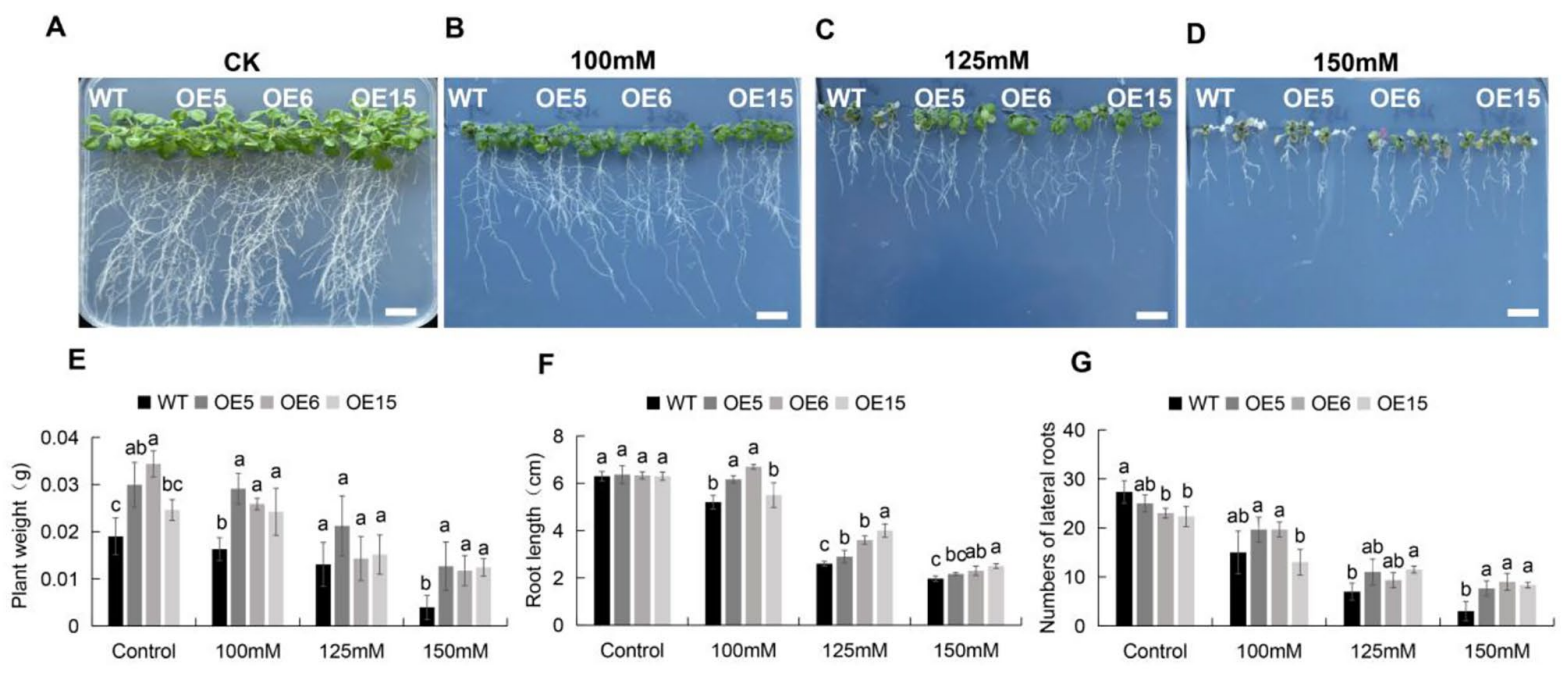
Salt tolerance analysis in seedlings grown on vertical plates for 15 days. (**A-D**) Images of wild-type (WT) and *MtEF1A1*-overexpression (OE) *Arabidopsis* seedling growth on vertical plates with media containing a range of salt concentrations (0–150 mM NaCl). Scale bar = 1 cm. CK, control (0 mM NaCl). Plant weight (**E**), root length (**F**), and lateral root number (**G**) of WT and *MtEF1A1*-OE seedlings grown on vertical plates with a range of salt concentrations (0–150 mM). Values are means ± SD of three biological replicates. Different lowercase letters between different OE lines of one treatment indicate significant differences (*p* < 0.05)

Fifteen *Arabidopsis* lines overexpressing *MtEF1A1* were generated. Three of these overexpression (OE) lines (OE5, OE6, and OE15) were randomly selected and *MtEF1A1* transcription was measured (Additional file 2: Fig. S3A). On media containing 0, 100, 125, or 150 mM NaCl, the *MtEF1A1*-OE lines exhibited significantly higher germination rates than the WT after 7 days. There were no significant differences in germination rates between WT and *MtEF1A1*-OE plants grown on agar plates without added salt (Fig. 2). WT, OE5, OE6, and OE15 plants were then grown on 1/2 Murashige and Skoog (MS) medium for 7 days, then transferred to vertical petri dishes containing 0, 100, 125, or 150 mM NaCl for 15 days to measure root growth. In salt-stressed plants, root growth inhibition was lower and leaf phenotypes were superior in the OE lines compared to the WT. Under the 150 mM NaCl treatment, the leaves of most *MtEF1A1*-OE plants remained green (Fig. 3D). In response to the range of salt stress conditions, the main root length was 6–54% higher in OE5, OE6, and OE15 than in WT seedlings. Without salt treatment, *MtEF1A1*-OE plants had fewer lateral roots than WT plants. However, as the salt concentration increased, OE plants had more lateral roots than the WT. The difference was significant at 150 mM NaCl, with the OE plants having 2.5–3 times as many lateral roots as WT plants. Plant weight was negatively correlated with the number of lateral roots without salt treatment (i.e., OE plants had heavier roots but fewer lateral roots than the WT). There was no significant difference in plant weight at 125 mM NaCl, but the plant weight was significantly higher for OE than WT plants at 100 mM NaCl and 150 mM NaCl (Fig. 3).

**Fig. 4:**
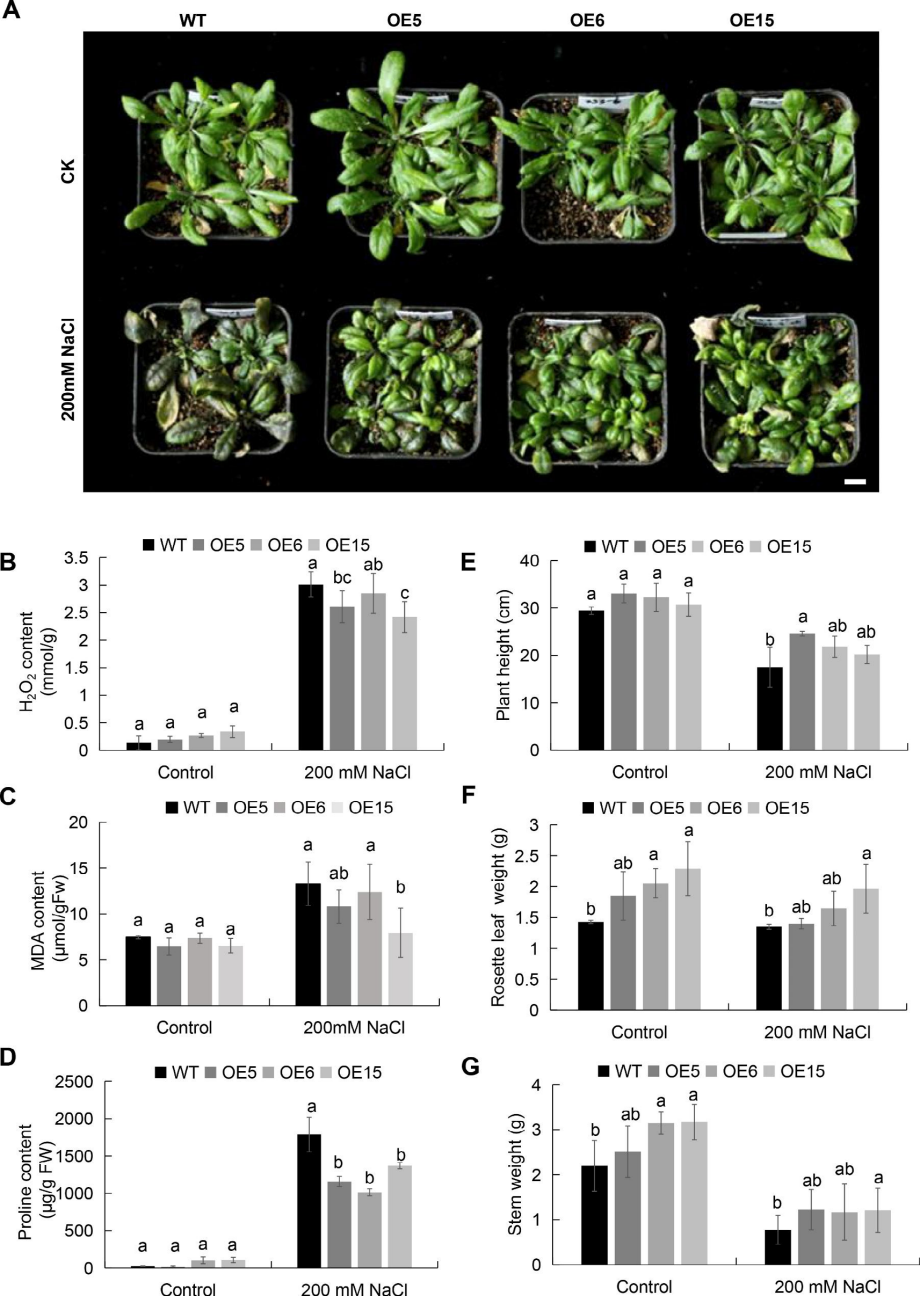
Salt tolerance analysis of three-week-old *Arabidopsis* seedlings grown in soil. (**A**) Phenotypes of three-week-old *MtEF1A1*-overexpression (OE) and wild-type (WT) *Arabidopsis* plants after salt treatment for 21 days. Scale bar = 1 cm. CK, control (0 mM NaCl). (**B-D**) Levels of H_2_O_2_, MDA, and proline in leaves. Plant height (**E**), rosette leaf weight (**F**), and stem weight (**G**) of WT and OE plants treated with 0 or 200 mM NaCl. Values are means ± SD of three biological replicates. Different lowercase letters between different OE lines of one treatment indicate significant differences (*p* < 0.05)

To further test the salt tolerance of *Arabidopsis MtEF1A1*-OE plants, three-week-old transgenic and WT plants grown in soil were treated with 0 or 200 mM NaCl for three weeks. There were no significant differences in leaf color between untreated WT and OE plants. However, with salt treatment, there were differences in growth and physiological indexes, with severe wilting and some chlorosis of WT rosette leaves (Fig. 4A). OE lines exhibited lower levels of H_2_O_2_ and MDA contents compared to the WT (Fig. 4B-C). Salt stress can lead to less accumulation of proline levels in the OE lines leaves (Fig. 4D). Specifically, OE15 seedlings accumulated 50% less MDA than WT seedlings, indicating that *MtEF1A1* overexpression enhanced salt tolerance in *Arabidopsis*. To explore the effects of salt treatment on *Arabidopsis* growth, the rosette leaf fresh weight, plant height, and stem weight were measured (Fig. 4E-G). Under untreated growth conditions, *MtEF1A1*-OE plants had higher height and weight than WT plants, but the differences were not significant in plant height. In salt-treated plants, WT plants showed greater growth inhibition with respect to rosette leaf weight and stem weight than *MtEF1A1*-OE plants, whereas the aboveground weight of OE15 was significantly higher than in WT plants after salt treatment.

### ***MtEF1A1*** overexpression reduced chlorophyll degradation in transgenic ***M. truncatula*** leaves

**Fig. 5:**
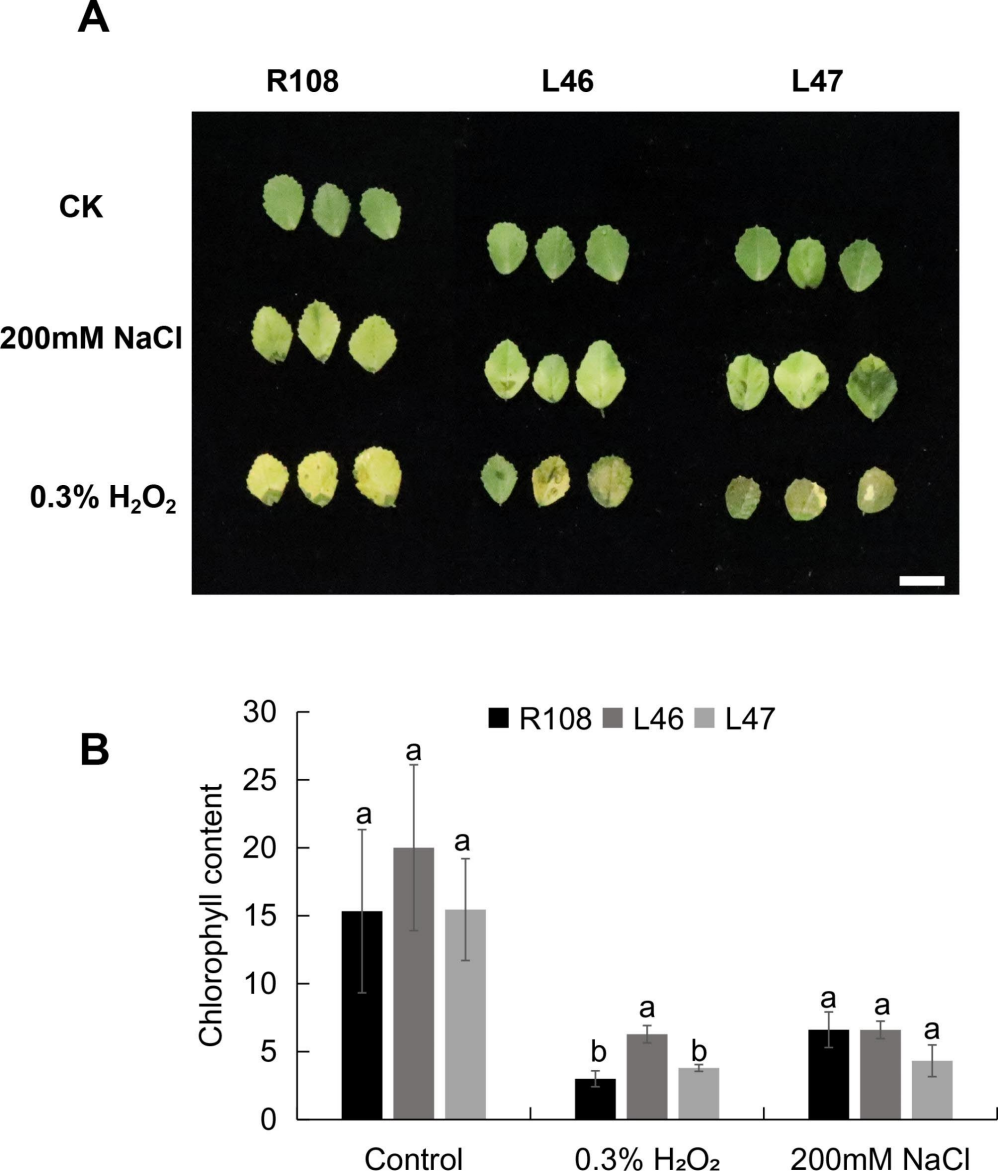
Leaf phenotypes of wild-type (WT) and *MtEF1A1*-overexpression (OE) *M. truncatula* lines treated with salt or H_2_O_2_. (**A**) Leaf phenotypes of WT (R108) and *MtEF1A1*-OE plants (L46 and L47). Plants were treated with 200 mM salt or 0.3% H_2_O_2_. Scale bar = 1 cm. CK, control (0 mM NaCl). (**B**) Leaf chlorophyll content in R108, L46 and L47 plants after salt or H_2_O_2_ treatment. Values are means ± SD of three biological replicates. Different lowercase letters between different OE lines of one treatment indicate significant differences within each treatment (*p* < 0.05)

Sixteen *MtEF1A1*-OE lines were next generated in *M. truncatula*, and two (L46 and L47) were selected for further study. The experiment was carried out using T_2_ individuals. *M. truncatula* R108 plants were used as the WT control. *MtEF1A1* expression was 23–32 times higher in L46/L47 than in R108 plants (Additional file 2: Fig. S3B). In untreated plants, there was no significant difference in chlorophyll content between *MtEF1A1*-OE and R108 plants (Fig. 5A and B). After salt treatment, L46, L47, and R108 plants showed no significant difference in leaf color or chlorophyll content. After 0.3% H_2_O_2_ treatment, R108 leaves were clearly yellow, and L46/L47 leaves gradually turn dark green or yellowish (Fig. 5A). The chlorophyll content was significantly decreased compared with the *MtEF1A1*-OE line L46 (Fig. 5B). These results indicated that *MtEF1A1* overexpression reduced toxic effect of ROS by H_2_O_2_ treatment of *M. truncatula* leaves in vivo.

### ***MtEF1A1*** overexpression improved salt tolerance in transgenic ***M. truncatula***

**Fig. 6:**
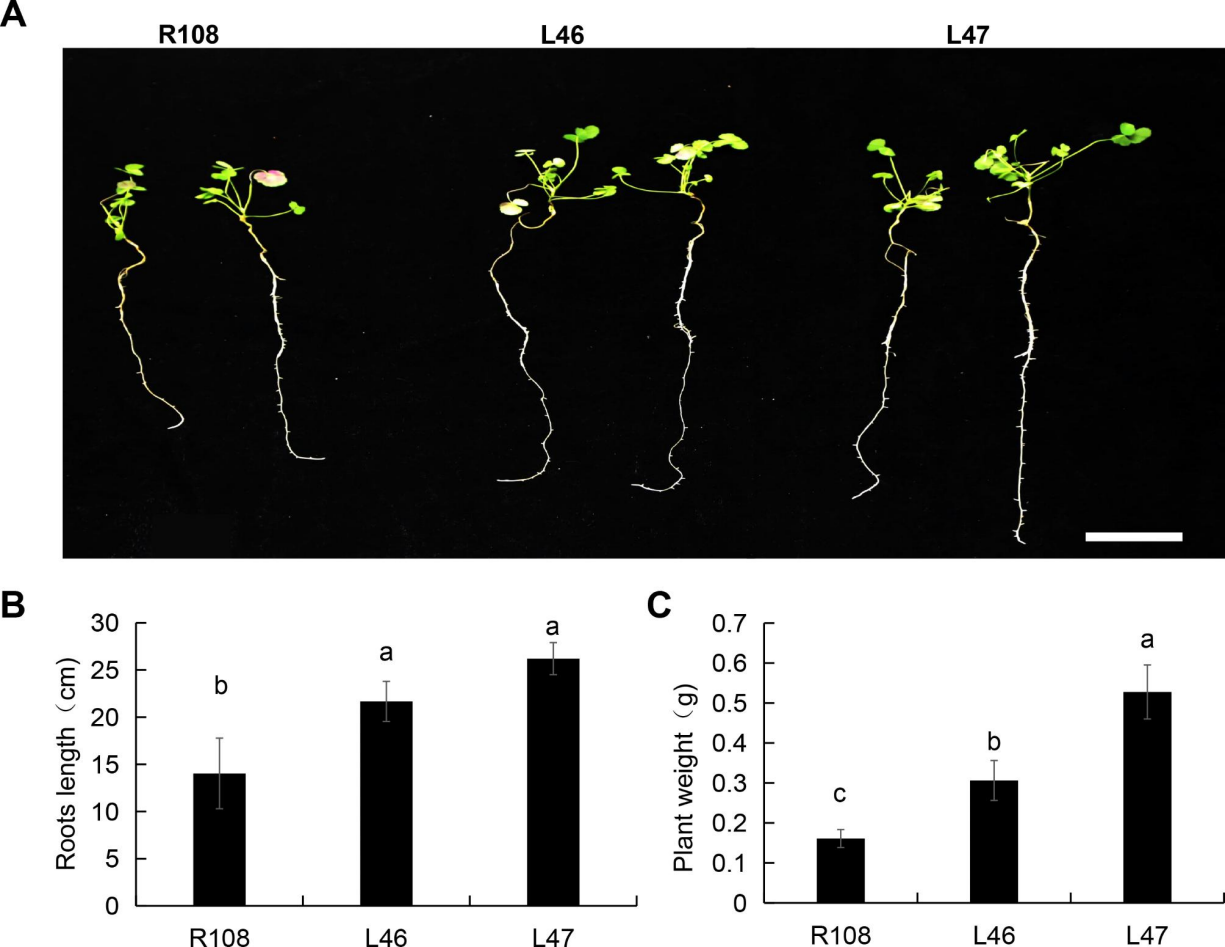
Salt stress analysis in three-week-old *M. truncatula* plants. (**A**) Images of wild-type R108 and *MtEF1A1*-overexpression (OE) plants (L46 and L47). Plants were cultured in 1/2 Hoagland nutrient solution with 100 mM NaCl for two weeks. Scale bar = 5 cm. (**B-C**) Roots length (**B**) and plant weight (**C**) of R108, L46 and L47 plants after salt stress. Values are means ± SD of three biological replicates. Different lowercase letters indicate significant differences (*p* < 0.05)

*M.truncatula* plants (R108 and EF1A1-OE lines) were cultured in 1/2 Hoagland nutrient solution medium for three weeks. Mature seedlings were collected and treated with 100 mM NaCl for two weeks. R108 plants gradually wilted and collapsed, and the abaxial surfaces of some leaves began to turn purple (Fig. 6A). L46 and L47 had longer roots than R108, and the difference was statistically significant (Fig. 6B). L46 and L47 also had significantly higher plant weight than R108 (Fig. 6C).

**Fig. 7:**
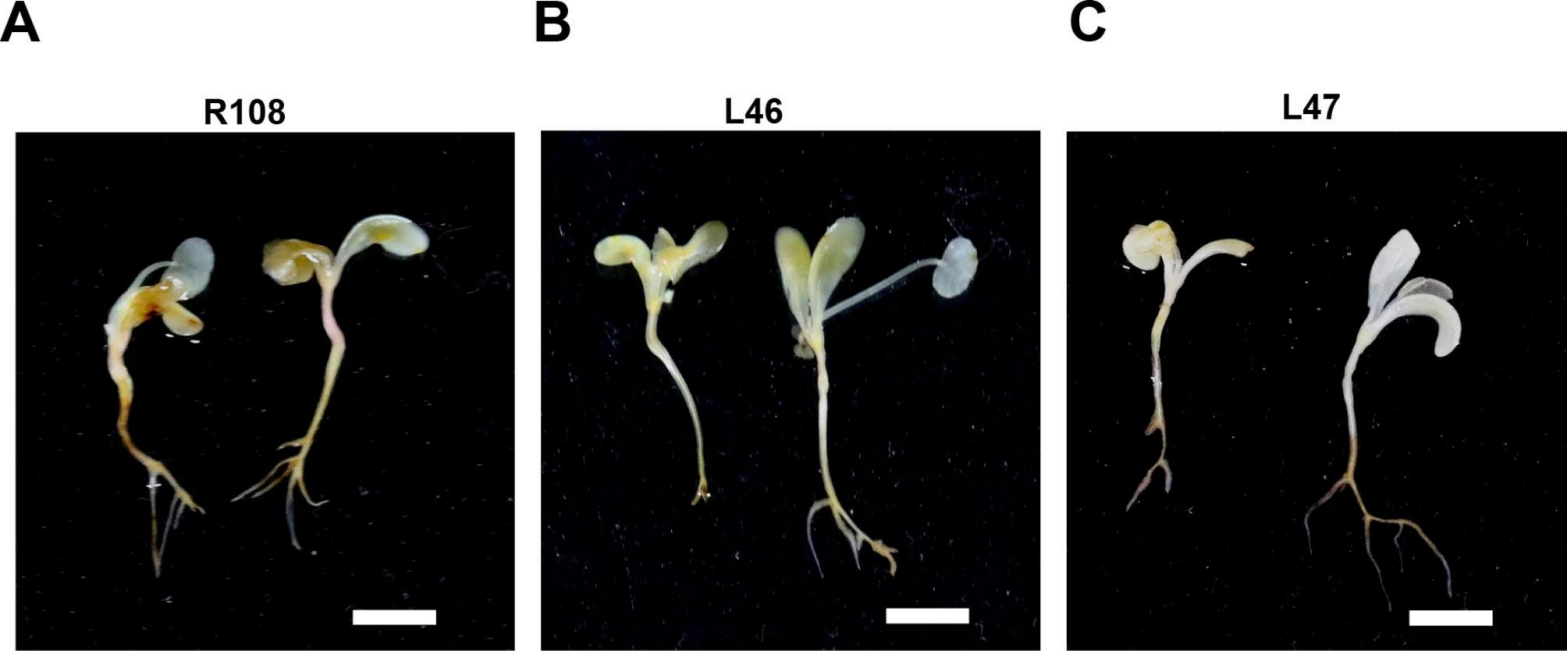
DAB staining assay in *M. truncatula* seedings treated with salt. (**A**) Wild-type (R108) and (**B, C**) *MtEF1A1*-overexpression (L46 and L47) seedlings were cultured in 1/2 MS medium with 150 mM NaCl. Scale bar = 1 cm

To explore the effect of *MtEF1A1* overexpression on salt stress tolerance at the seedling stage of *M. truncatula*, plants treated with salt for 10 d were stained with DAB to detect accumulation of H_2_O_2_. Observation of whole seedlings stained with DAB showed that R108 had a much larger stained area and stained darker than L46 and L47 plants, indicating higher levels of ROS in R108 (Fig. 7).

### Effects of ***MtEF1A1*** overexpression on abiotic stress-responsive gene expression

**Fig. 8:**
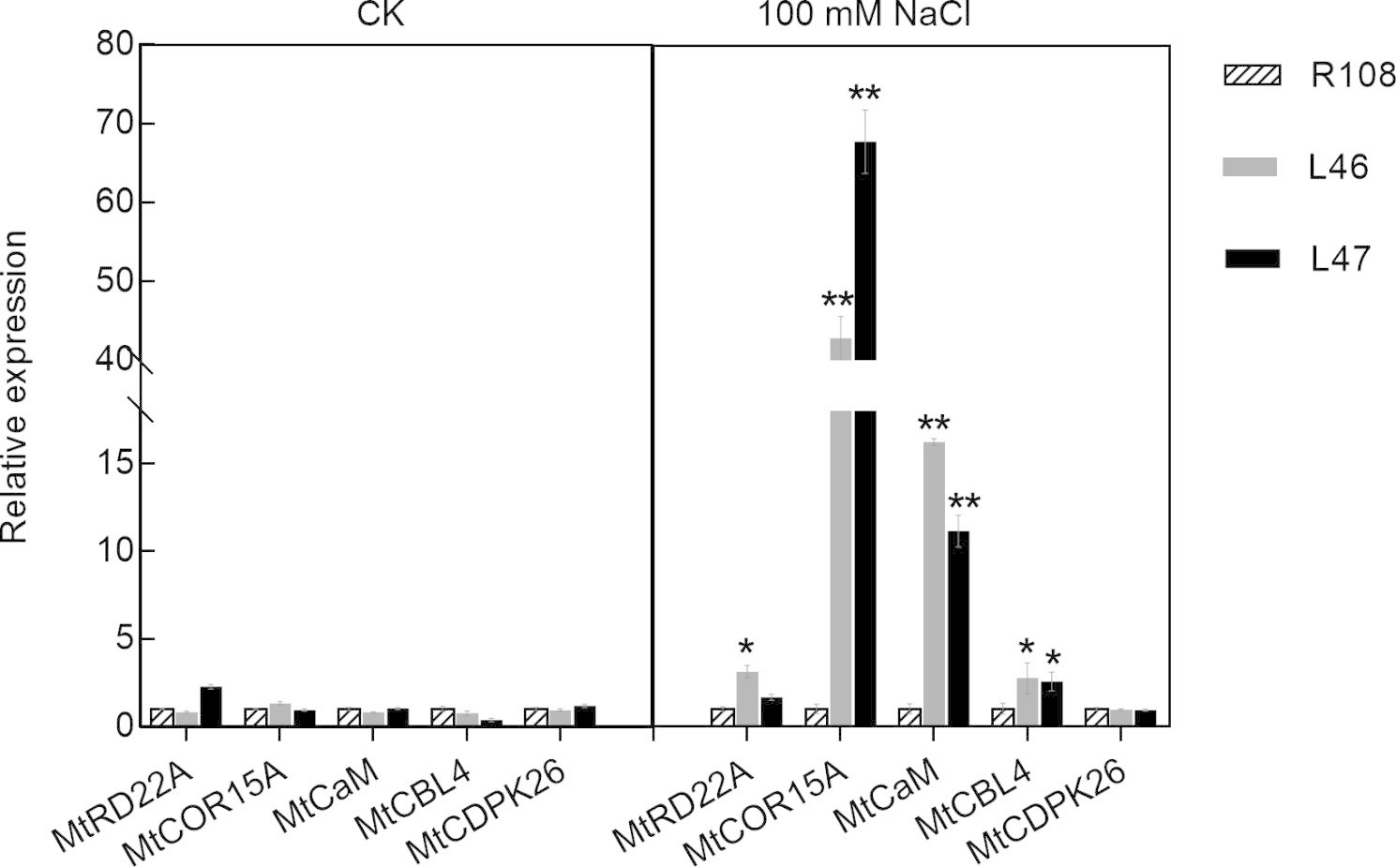
Expression of stress-related genes of three-week-old *M. truncatula MtEF1A1*-overexpressing plants (L46 and L47) and wild-type plants (R108) in 1/2 Hoagland medium (CK) and 100 mM NaCl for two weeks. R108 was used as the reference to calculate relative expression levels in the overexpression lines. Values are means ± SD of three biological replicates. Different lowercase letters between different OE lines of stress-related genes indicate significant differences (*p* < 0.05). ** indicates a significant difference at *p* < 0.01, * indicates a significant difference at *p* < 0.05

**Fig. 9:**
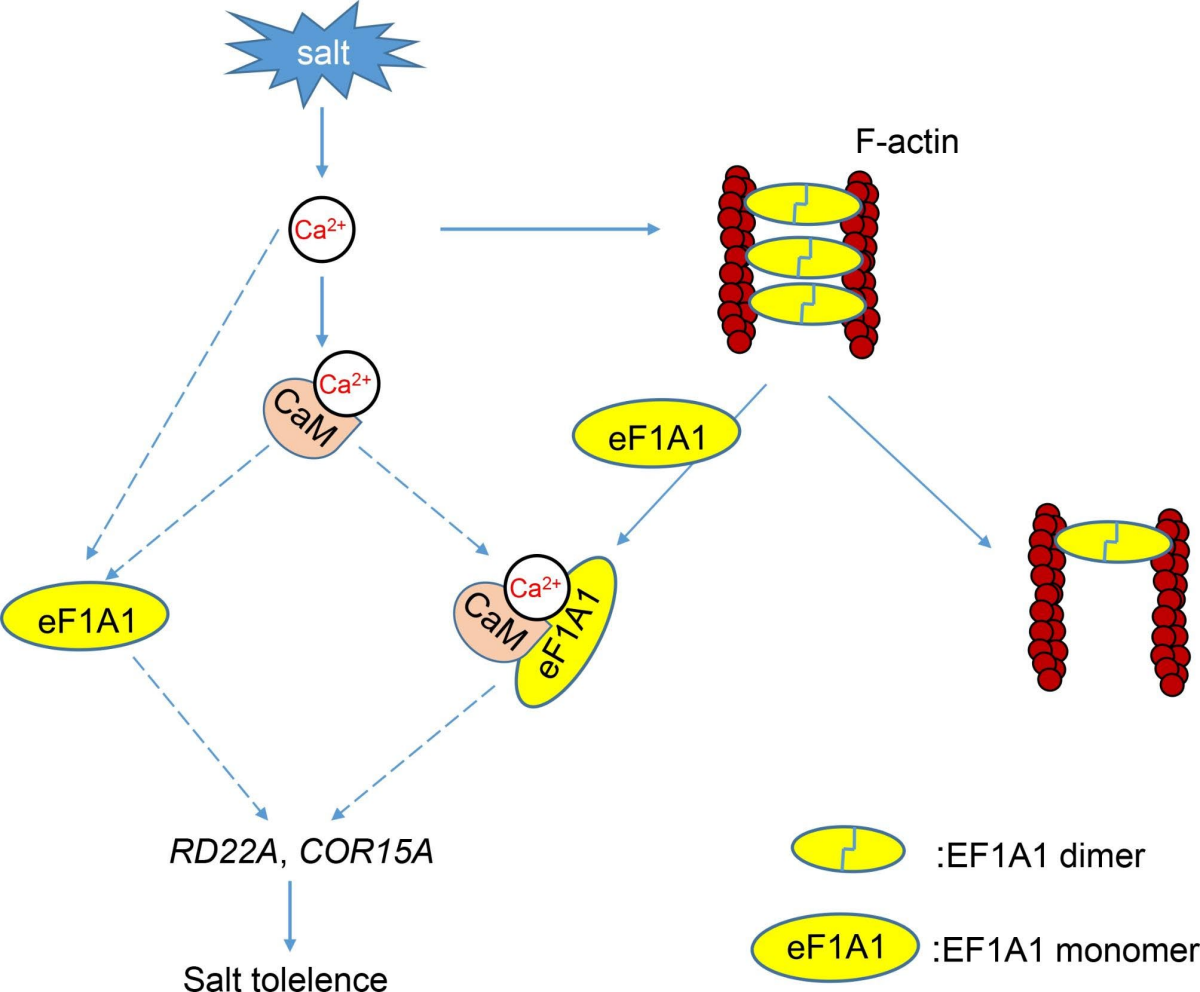
Proposed model of the regulatory mechanism of *MtEF1A1* in response to salt stress. Salt stress induces the expression of *MtEF1A1* through Ca^2+^ signaling. *MtEF1A1* promotes expression of calmodulin (CaM), which binds Ca^2+^ to promote the expression of downstream genes, *MtEF1A1*, or the eEF1A1/Ca^2+^/CaM complex

To determine a possible mechanism for *MtEF1A1* functioning in the salt stress response, expression levels of known stress-responsive genes were analyzed in R108 and *MtEF1A1*-OE *M. truncatula* plants after salt treatment. *BURP domain protein RD22* (*MtRD22A*), *Cold-regulated 15 A* (*MtCOR15A*), *Calmodulin* (*MtCaM*) and *Calcineurin B-like proteins 4* (*MtCBL4*) were upregulated in *MtEF1A1*-OE plants compared to R108, and *MtCOR15A* and *MtCaM* genes were significantly induced after salt treatment. In untreated *M. truncatula* (CK), there were no significant differences in these genes (Fig. 8). We therefore speculate that *MtEF1A1* may be involved in Ca^2+^-dependent signaling pathways that are induced by salt stress (Fig. 9). CaM participates in Ca^2+^-dependent signaling pathways associated with stress and promotes dimerization of target proteins through direct binding. However, there was no significant difference in the expression of *Calcium-dependent protein 26* (*MtCDPK26*) between the R108 and *MtEF1A1*-OE lines (Fig. 8).

## Discussion

Elongation is the most important step in translation and is completed by highly conserved eukaryotic *EF* genes. EF1A protein has chaperone activity and can function in protein renaturation and structural changes [32]. The EF1A plastid protein is encoded by the nuclear genome, synthesized in the cytoplasm, then targeted to the plastid. EF1A is also found in mitochondria, which can play a role in protein synthesis and translation by catalyzing the extension of amino acid chains on the ribosome [33]. The sequence of *MtEF1A1* (MTR_6g021805) was obtained from Ensembl (http://plants.ensembl.org/index.html), and homology comparison on the TAIR website (https://www.arabidopsis.org/cgi-bin/Blast/) yielded the four *Arabidopsis* genes with the highest similarity (96% amino acid sequence identity): *AtEF1A1* (*AT1G07920*), *AtEF1A2* (*AT1G07930*), *AtEF1A3* (*AT1G07940*), and *AtEF1A4* (*AT5G60390*). *EF1A* plays a role in protein synthesis, and it is often considered a housekeeping gene due to its conserved structure between different species. However, plant EF1A proteins are always encoded by multiple genes, and *EF1A* expression is not consistent between organs and developmental stages. In tomato and apple, *EF1A* is expressed at higher levels in young leaves than in mature leaves [34, 35]. *EF1A* is more highly expressed in the stems and roots of Chinese jujube (*Ziziphus jujube*) and cotton (*Gossypium* spp.) compared to the flowers [36, 37], but the opposite is true in banana (*Musa paradisiac*a) [38]. *EF1A* is expressed at higher levels in tomato roots than stems [39], consistent with the expression of *MtEF1A1* in *M. truncatula* (Fig. 1C).

The growth status of plants is shown by related characteristics indicators such as plant height, plant weight, root length and lateral root number, which can be inhibited under stress conditions. At the same time, stress can affect indicators of plant growth at different stages, such as germination rate and survival rate. Studies have shown involvement of *EF1A* in abiotic and biotic stress responses in plants, potentially allowing for crop improvement via manipulation of *EF1A* [33]. In the present study, *Arabidopsis* plants overexpressing *MtEF1A1* showed that there was no significant difference in terms of germination rate compared with WT plants when they were grown on 1/2 MS medium; *MtEF1A1*-OE lines did have a significantly higher germination rate than WT when the seedlings were exposed to salt stress (Fig. 2). This indicated that overexpression of *MtEF1A1* could improve germination rate to some extent. WT *Arabidopsis* plants were more sensitive to salt stress than OE lines; the germination rates of OE5, OE6, and OE15 plants were higher than the WT (Fig. 2A and D). In *Arabidopsis* plants grown in soil, those overexpressing *MtEF1A1* had higher weight than WT plants, with or without salt treatment (Fig. 4A); there was also a significant difference in the stem weight of *MtEF1A1*-OE plants (Fig. 4G). Based on these results, *MtEF1A1* may be involved in pathways related to plant salt stress responses, but may also improve growth and development of *Arabidopsis*. It has previously been shown that there is crosstalk pathways related to salt stress responses and plant development [40]. The content of MDA, H_2_O_2_, proline and chlorophyll in plant leaf cells is an important physiological index to evaluate plant stress resistance, which can essentially explore the physiological mechanism of plants. In soybean, the contents of H_2_O_2_, O_2_^−^ and MDA decreased, while the content of proline increased in *GmEF4* overexpressing plants after stress treatment [29]. H_2_O_2_ is an important reactive oxygen molecule. In this study, the OE lines maintained lower levels of H_2_O_2_ and the result showed that OE lines could decrease the membrane lipid peroxidation damage caused by stress (Fig. 4B) [29]. The MDA content is known as a physiological marker of lipid peroxidation rate in plant, and is correlated with oxidative stress [41]. MDA was lower in OE lines indicating that the plasma membrane of OE lines was less influenced and lipid peroxidation was reduced (Fig. 4C). Proline, a stress response factor, did not always accumulate significantly in plants under stress, and the proline content of OE lines was lower than WT, which may be that the proline accumulation at this point appears to be a response to injury rather than an alleviator, and *MtEF1A*-overexpression *Arabidopsis* lines were less stimulated by the stress than WT [42].

Similar results were observed in *M. truncatula*; *MtEF1A1* overexpression improved salt tolerance. To verify the salt tolerance of three-week-old *M. truncatula* plants, we examined the responses of WT and *MtEF1A1*-OE plants to salt stress during seedling growth (Fig. 6). Under salt stress conditions, *MtEF1A1*-OE lines had longer roots and higher fresh weight compared with the WT plants significantly (Fig. 6A-C). However, the difference of root length was not significant between the two OE lines, but there was difference in plant weight (Fig. 6B and C). This may have been a result of the low contribution of root weight to the total fresh weight. The influence of *MtEF1A1* overexpression on aboveground tissue weight compared to underground biomass requires further study in *M. truncatula*. In *M. truncatula* grown in soil for 60 d, those overexpressing *MtEF1A1* isolated leaves had increased tolerance of H_2_O_2_ treatment, and the chlorophyll content was significantly higher in the leaves of one *MtEF1A1*-OE line than in WT plants. However, when isolated leaves were treated with 200 mM NaCl solution, there was no difference in chlorophyll content between *MtEF1A1*-OE and WT leaves (Fig. 5). This may be due to the faster process of ROS accumulation after H_2_O_2_ treatment. Meanwhile, salt treatment of the whole *Arabidopsis* also affected the H_2_O_2_ content of the plant, which *EF1A* overexpression can alleviate (Fig. 4B). DAB staining in seedlings showed that the *MtEF1A1*-OE lines had lower ROS accumulation in response to salt stress compared to the WT, and that the WT showed generally higher ROS accumulation in the roots. This may be due to the high expression level of *EF1A* in the roots of *M. truncatula*, so the root damage degree of *EF1A*-overexpression lines was lower than that of WT under salt stress (Fig. 7). All above experiments of stress treatment in isolated leaves and DAB staining test speculated that overexpression of *MtEF1A1* may enhance plant salt tolerance by inhibiting the accumulation of ROS.

Regulation of salt stress involves the salt sensitive (SOS) pathway, which regulates a range of proteins, including kinases, ion transporters [43], members of the calcium-dependent protein kinase (CDPK) pathway [44], and those in the mitogen-activated protein kinase (MAPK) pathway [45]. Ca^2+^ signaling usually functions as a secondary messenger in response to external stimuli. CaM, CAM-like protein (CML), and CDPKs regulate plant resistance by binding to downstream effector proteins. For example, *AtCaM7* acts as a transcription factor in direct response to light stress [46]. *AtTGA3* transcription factor can directly bind to the *AtCaM3* promoter region and promote downstream stress response [47]. Ca^2+^ signal-dependent CaM binding usually causes structural changes in the target proteins or promotes their expression [48–50]. *MtCDPK26* and *AtCPK6* are both CDPKs. *AtCPK6* and other CDPKs are activated by Ca^2+^ signals. *AtCPK6* overexpression promotes salt stress tolerance in *Arabidopsis* [51, 52]. *Calcineurin B-like proteins* (CBLs) act as Ca^2+^ sensors; their targets, *CBL-interacting protein kinases* (CIPKs), function in plant signal regulation. Salt signaling promotes Ca^2+^ accumulation, which activates CBL4/SOS3 and CBL10 Ca^2+^ sensors to interact with CIPK24/SOS2 and participate in ion transport with SOS1 [53]. *AtRD22* and *AtCOR15A* can participate in abscisic acid (ABA)-dependent pathways, and are upregulated by a variety of abiotic stressors. *AtCOR15A* has DRE and other abiotic stress-associated motifs in the promoter and participates in the *Arabidopsis* CBF-related cold resistance pathway [54]. *AtRD22* is believed to be primarily regulated by activation of *AtMYB2/AtMYC2* expression [55]. In the present study, we found that *CaM* expression was significantly increased in response to salt treatment of *EF1A* overexpression in *M. truncatula*. However, there were no significant differences in expression of *MtCDPK26*, suggesting that *MtEF1A1* may interact with *MtCaM* but that it does not directly participate in regulation of CDPK pathway.

Previous studies showed that *EF1A* genes may be involved in transcription or post-transcriptional regulation in response to salt stress [25]. As a multifunctional protein, EF1A is also highly sensitive to changes in Ca^2+^ and pH, and may be an important downstream target of Ca^2+^ and lipid-related signal transduction pathways, causing changes in cytoskeleton structure and protein synthesis [56]. EF1A may not only participate in the common salt response pathway, but may also function at the protein level. EF1A has been shown to interact directly with tubulin, calmodulin, and filamentous actin (F-actin) in vitro [57]. EF1A has two forms, a monomer and a dimer. Under normal circumstances, the EF1A dimer bundles into F-actin to form a binding protein. In the presence of salt or high levels of Ca^2+^, the EF1A/Ca^2+^/CaM complex binds to F-actin, and EF1A in the dimer form reverts to the monomeric state [58, 59]. The eEF1A/Ca^2+^/CaM complex is speculated to regulate the downstream salt response pathway either directly or indirectly (Fig. 9). Protein hydrolysis can rapidly reduce the functional activity of EF1A. Treatment with phospholipase C and D leads to the reduction of amount of [^14^ C] ethanolamine-labeled eEF1A in the carrot microsomal fraction, and EF1A content can be quantified by PGE modification [56]. Studying the dynamic changes of *MtEF1A1* levels in response to stress treatment is expected to provide novel insights into the salt-response pathway of EF1A.

## Conclusion

Elongation factors are indispensable in protein synthesis. Overexpression of the elongation factor *MtEF1A1* in *Arabidopsis* enhanced salt resistance, increasing the seed germination rate and decreasing levels of H_2_O_2_, MDA, and proline in the leaves of *MtEF1A1*-OE plants compared to the WT. In *M. truncatula* overexpressing *MtEF1A1*, leaf chlorophyll level was higher than in WT leaves after treatment with H_2_O_2_. Root damage was more severe in WT seedlings and DAB staining was darker, indicating higher ROS levels. The *MtEF1A1* of *M. truncatula* plants were resistant to salt stress and showed higher expression of the Ca^2+^-signaling-related genes *MtCaM* and *MtCBL4*, and the salt-induced genes *MtRD22A* and *MtCOR15A*. This study shows that overexpression of *MtEF1A1* increased salt stress tolerance in *Arabidopsi*s and *M. truncatula*, indicating that *MtEF1A1* plays a positive regulatory role in the salt stress response.

## Materials and methods

### Plant materials and growing conditions

*Arabidopsis* Col-0 used in this study was provided by our lab. Seeds were stratified for 2 d under dark conditions at 4 °C, then cultured on 1/2 MS medium. After 7 d, the seedlings were transferred to petri dishes in incubator under a 16/8 h light/dark photoperiod at 22/18°C, or transferred to soil prior to salt treatments in a greenhouse (26/22°C, 16/8 h light/dark photoperiod).

For *M. truncatula* experiments, ecotype R108 provided by our lab was used as the WT. Seeds were surface-sterilized and grown on 1/2 MS medium at 22/18 ℃ under a 16/8 h light/dark cycle in a growth chamber with 60–70% relative humidity. *M. truncatula* seeds germinated on wet filter paper were transferred to 1/2 Hoagland’s solution and grown in a growth chamber, or transferred into soil (1:1 vermiculite:sand) and grown in a greenhouse at 26/22°C under a 16/8 h light/dark cycle.

### Cloning ***MtEF1A1*** and bioinformatics analyses

A GTP-binding factor Tu family protein gene (*MTR_6g021805*) from *M. truncatul*a was cloned in our study. BLASTP results showed that *MTR_6g021805* shared high similarity (96%) and a conserved functional domain with *AtEF1A1* (AT1G07920); *MTR_6g021805* was therefore designated *MtEF1A1*. Primer Premier 5.0 was used to design primers to clone the CDS of *MtEF1A1* (Additional file 1: Table S1).

ProtParam on ExPASy (https://web.expasy.org/protparam/) was used to predict the isoelectric point and molecular weight of MtEF1A1; Prosite (https://prosite.expasy.org/) and Protscale were used to analyze the protein domains, conserved structures, and protein hydrophobicity (https://web.expasy.org/protscale/) [60]. SOPMA (https://npsa-prabi.ibcp.fr/cgi-bin/npsa_automat.pl?page = npsa_sopma.html) was used to analyze secondary protein structure [61]. Tertiary protein structure was analyzed with SWISS-MODEL (https://swissmodel.expasy.org/interactive) [62]. Protein transmembrane structure and signal peptide predictions were performed with Phobius (http://phobius.sbc.su.se/) [63]. Protein subcellular localization was predicted with Cell-PLoc 2.0 (http://phobius.sbc.su.se/) [64].

*MtEF1A1* sequences and *EF1A* homologs in *Arabidopsis*, soybean, and rice were obtained from ensemblplant and aligned using DNAMAN software to determine the functional domains. The evolutionary relationships between *EF1A* genes were determined with ITOL online software. Clustal Omega was used to perform multiple sequence alignment of *M. truncatula* EF1A proteins. Motifs in the *M. truncatula* EF family were identified with MEME (http://meme-suite.org/).

### ***MtEF1A1*** gene expression

Total RNA was extracted using the Eastep Total RNA Extraction Kit (Promega, Beijing, China), and cDNA was synthesized using the PrimeScript™ RT Reagent Kit with gDNA Eraser (Takara, Shiga, Japan). cDNA was diluted to 100 ng/µL. qRT-PCR was performed using TB Green® Premix Ex Taq™ II (Tli RNaseH Plus) (Takara, Shiga, Japan) on the CFX384 Touch Real-time PCR Detection System (Bio-Rad, Hercules, CA, USA). Each 20 µL qRT-PCR reaction contained 10 µL 2x SYBR Premix Ex Taq mix, 0.2 mM of each primer, and 2 µL cDNA. Primers for each gene are shown in Table S1. *MtActin* and *AtActin* were used as the internal reference genes for *M. truncatula* and *Arabidopsis* samples, respectively. Relative gene expression was calculated using the 2^−ΔΔCt^ method [65]. There were three technical replicates for each experiment.

### Transformation of ***Arabidopsis*** and ***M. truncatula***

The CDS of *MtEF1A1* was inserted into the plant transformation vector pCAMBIA3301 driven by the CaMV 35 S promoter. The *35 S::MtEF1A1-GUS* expression plasmid was transferred into *Agrobacterium tumefaciens* strain GV3101. *Arabidopsis* plants were then transformed using the floral dip method [66]. The seeds of transformed plants were cultured on 1/2 MS medium and inoculated with 4 mg/L phosphinothricin (PPT) (Sigma-Aldrich, St Louis, MO, USA) for 7 d. Homozygous T_3_ plants were obtained for use in further experiments. *Agrobacterium* strain EHA105 with an overexpression vector was used to infect *M. truncatula* using the leaf infection method [67]. Well-developed transgenic *M. truncatula* seedlings screened with 2 mg/L PPT were transplanted into soil, identified with gene-specific primers, and T_0_ seeds were collected. Homozygous T_2_ plants were used for subsequent experiments.

### Salt tolerance analyses in ***Arabidopsis***

Transgenic and WT *Arabidopsis* seeds were disinfected, then incubated for 7 d on 1/2 MS medium with 0, 100, 125, or 150 mM NaCl. Germination rates were recorded for each treatment. Transgenic and WT *Arabidopsis* seedlings were grown in 1/2 MS medium for 7 d, then transferred to vertical petri dishes containing 1/2 MS with 0, 100, 125, or 150 mM NaCl for 15 d. The fresh weight, root length, and lateral root number were then measured. Three-week-old *Arabidopsis* seedlings grown in soil under cool-white fluorescent lights were treated with 0 or 200 mM NaCl solution every 3 d for a period of 21 d. Plant phenotypes were recorded after stems were removed and rosette leaves were collected, immediately frozen in liquid nitrogen, and stored at 80 °C prior to further analysis. There were three replicates for each treatment.

### Salt tolerance analyses of ***M. truncatula***

Four-week-old WT *M. truncatula* (R108) seedlings grown in 1/2 Hoagland medium were incubated in 200 mM NaCl. Roots, stems, and leaves were collected at 0, 1, 2, 8, 12, and 24 h. The experiment was repeated three times.

*MtEF1A1*-OE and WT plants were transplanted to 1/2 Hoagland nutrient solution for 3 weeks, then treated with 100 mM NaCl on 1/2 Hoagland’s solution for 2 weeks. The nutrient solution was changed every 5 d. Phenotypes were observed and the fresh weight and root length were measured. For *M. truncatula* leaf treatments, 60-day-old seedings grown in soil were treated with 30 mL distilled water, 200 mM NaCl, or 0.3% H_2_O_2_. After 5 d of treatment, leaf phenotypes were recorded. Chlorophyll levels were measured using the ethanol immersion method as described by Emel Ergun [68].

### Physiological indices quantification and DAB staining

H_2_O_2_, malondialdehyde (MDA), and proline levels were measured in the leaves of transgenic and WT *Arabidopsis* seedlings using the Hydrogen peroxide determination kit (Jiancheng, Nanjing, China), the Micro Malondialdehyde (MDA) Assay Kit (Solarbio, Beijing, China), and the Proline (PRO) Content Detection Kit (Solarbio, Beijing, China), respectively. *M. truncatula* seeds (WT and *MtEF1A1-*OE lines) were grown on 1/2 MS medium for 2 d, then transferred to vertical plates on 1/2 MS supplemented with 150 mM NaCl. Seedlings were collected after 10 d and stained with 3,3’-dia-minobenzidine (DAB, Solarbio, Beijing, China). The stain (1 mg/ml) was placed in conical flasks containing seedlings; leaves were lowered below the liquid surface with vacuuming, then incubated in the dark for 5 h. After staining, fixative solution (3:1:1 ethanol:lactic acid:glycerol) was added and samples were boiled in water for 5 min. After cooling, leaves were rinsed with ethanol several times. Samples were observed with a Stemi microscope (ZEISS, Germany) and images were captured.

### Statistical analysis

All experiments were conducted with three biological replicates. Data were analyzed using analysis of variance (ANOVA) with post-hoc Duncan’s multiple range test for multiple comparisons.

## Electronic supplementary material

Below is the link to the electronic supplementary material.

Supplementary Material 1

## Data Availability

The genomic data of *M. truncatula* in the article can be downloaded from Phytozome (https://phytozome.jgi.doe.gov/pz/portal.html). Protein sequences of plant species includes *Arabidopsis thaliana*, *Glycine max*, and *Oryza sativa* were downloaded from EnsemblPlants database (http://plants.ensembl.org/index.html).

## Declaration of legitimacy

In this study, we comply with the IUCN Policy Statement on Research Involving Species at Risk of Extinction and the Convention on the Trade in Endangered Species of Wild Fauna and Flora. The procedures complied to the relevant institutional, national, and international guidelines and legislation.

## Abbreviations

MDA: Malondialdehyde
ROS: Reactive oxygen species
CaM: Calmodulin
EF: Elongation factors
GEF: Guanine nucleotide exchange factor
GST: Glutathione transferase
eEF2K: Extension factor-2 kinase
PPT: Phosphinothricin
DAB: 3,3’-dia-minobenzidine
RD22A: BURP domain protein RD22
COR15A: Cold-regulated 15 A
CBL4: Calcineurin B-like proteins 4
CDPK26: Calcium-dependent protein 26
MAPK: Mitogen-activated protein kinase
CIPKs: CBL-interacting protein kinases
F-actin: Filamentous actin.
