# Supplementary material for: Overexpression of the elongation factor MtEF1A1 promotes salt stress tolerance in Arabidopsis thaliana and Medicago truncatula
Source: BMC Plant Biol. 2023 Mar 13;23:138. doi: 10.1186/s12870-023-04139-5 (PMC10009949; doi:10.1186/s12870-023-04139-5)
Supplement: Supplementary file 1 — Supplementary Material 1 [file 12870_2023_4139_MOESM1_ESM.docx]

**Table S1.** Primers used in this study

| **Primer Name** | **Primer sequences** |
| --- | --- |
| *MtEF1A1*-F | GCATATTCCATCCGCCACT |
| *MtEF1A1*-R | CCATCGCCGATCAAGAAC |
| *Mt-Actin*-F | CAAAAGATGGCAGATGCTGAGGAT |
| *Mt-Actin*-R | CATGACACCGGTATGACGAGGTCG |
| *At-Actin*-F | GAAATCACAGCACTTGCACC |
| *At-Actin*-R | AAGCCTTTGATCTTGAGAGC |
| qRTPCR *MtEF1A1*- F | AGCTGCTGCCAAGAAGAAGT |
| qRTPCR *MtEF1A1*- R | CAAAAACGGTACCATCGCCG |
| qRTPCR *MtRD22A* F | ACCCCCTGAAGTGTACTGGA |
| qRTPCR *MtRD22A* R | CTGCTGTTCCACCTGGCTTT |
| qRTPCR *MtCOR15A* F | GTGTTGCAGCTGGTAGTCCT |
| qRTPCR *MtCOR15A* R | TCCGGTTGCATTAGCGAAGT |
| qRTPCR *MtCaM* F | TCCAGTCTCTCCGCATCTGA |
| qRTPCR *MtCaM* R | CCCAGTCATTTCAGGCCCAT |
| qRTPCR *MtCBL4* F | TTTGATGTCAAGCGCAACGG |
| qRTPCR *MtCBL4* R | TCCCCAGTTAGTCCCCTCTC |
| qRTPCR *MtCDPK26* F | CTCTACACTCTTGGCCGCAA |
| qRTPCR *MtCDPK26* R | GAGCATCCTCATAGGCACCC |

*
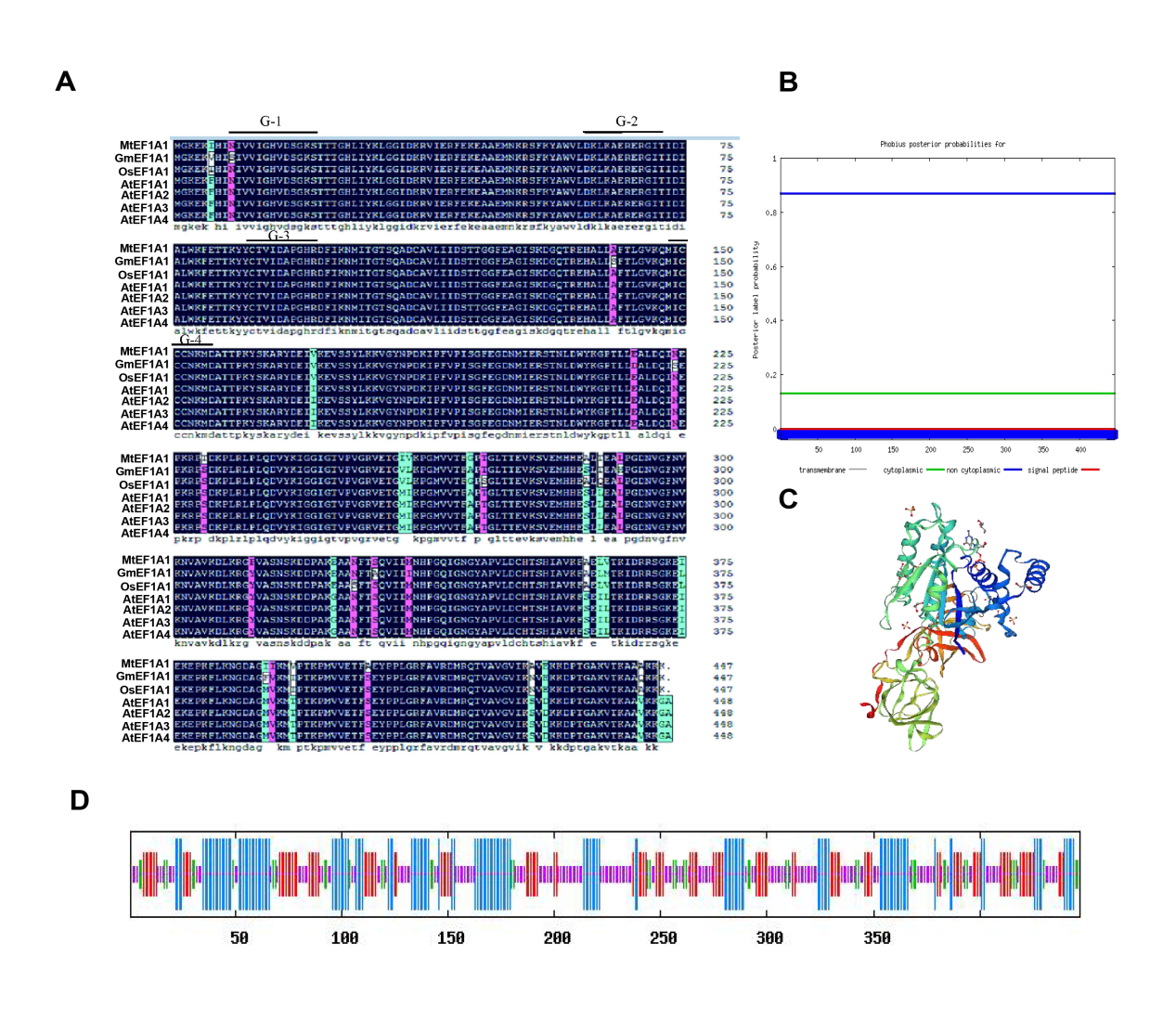
*

**Fig. S1.** Characteristics of MtEF1A1 and its homologous genes in three other species. (A) Comparison of MtEF1A1 with other EF1A protein sequences: *MtEF1A1* (MTR_6g021805), *GmEF1A1* (GLYMA_17G186600), *OsEF1A1* (Os03g0177400), and *AtEF1A1*-*4* (AT1G07920, AT1G07930, AT1G07940, AT5G60390). The conserved regions, namely G-1 (N9–S21), G-2 (D61–T72), G-3 (C87–R96), and G-4 (I149–D156), are involved in GDP/GTP exchange and GTP hydrolysis. (B) Protein transmembrane structure and signal peptide. (C) Tertiary and (D) secondary protein structure of MtEF1A1.


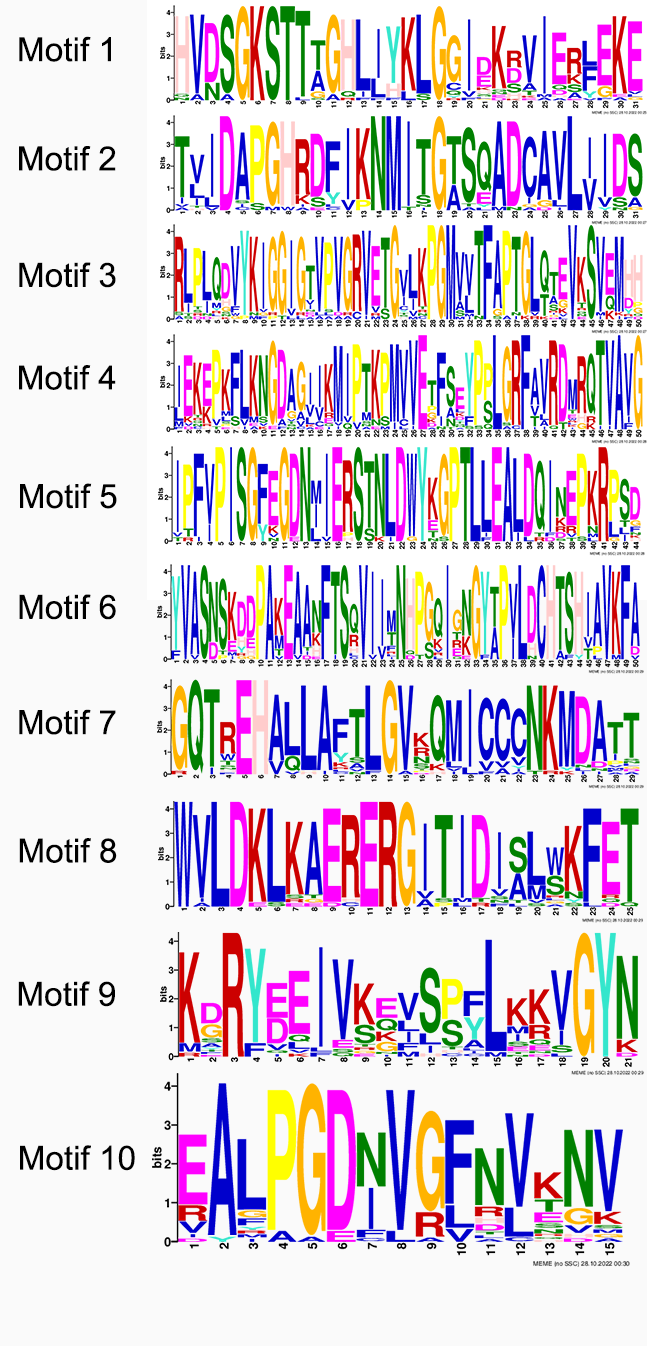


**Fig. S2.** Expression Logo of motifs in *EF* genes in *M. truncatula.* All 34 EF1 gene family members motif of *M. truncatula* were analyzed from the amino acid sequence using MEME.

**
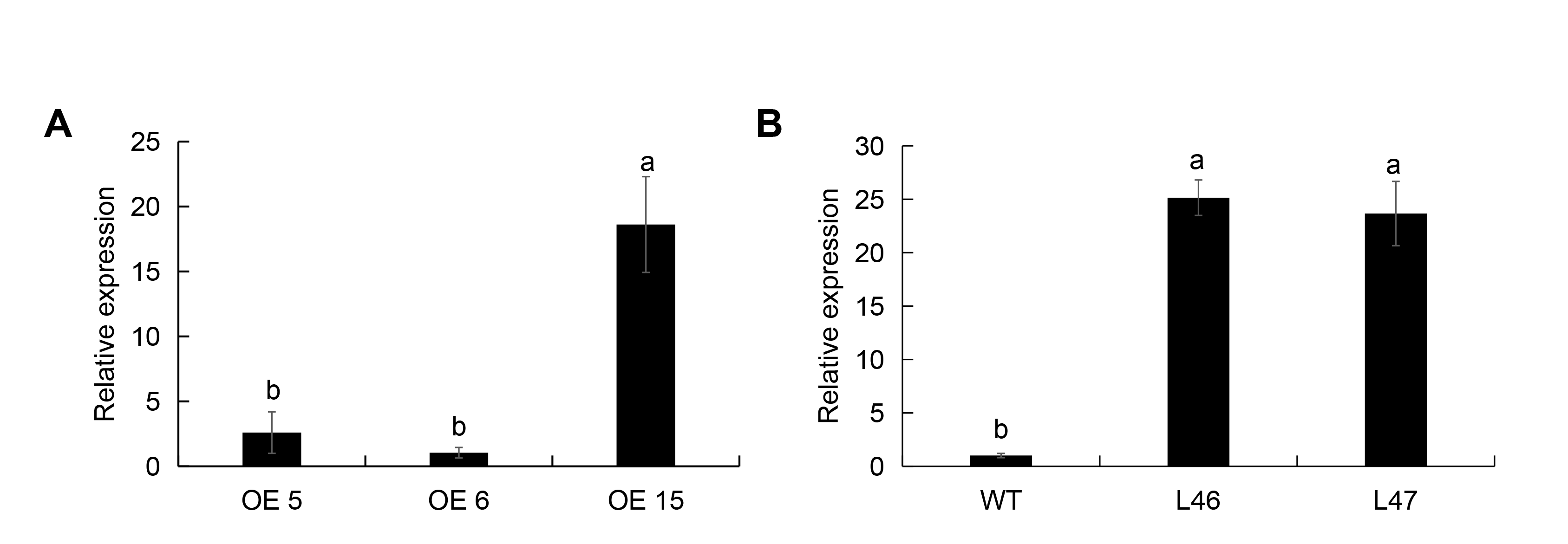
**

**Fig. S3.** Expression analysis of *MtEF1A1*. (A) Relative expression of *MtEF1A1* in transgenic *Arabidopsis* lines. OE6 was used as a reference to calculate relative expression in the other two OE lines. (B) Relative expression of *MtEF1A1* in transgenic *M. truncatula* overexpressing *MtEF1A1*. The wild-type line R108 was used as a reference to calculate relative expression in the other lines. Different lowercase letters indicate significant differences (*p* < 0.05).
